# Human and mouse seeds differentially affect Aβ and tau aggregation in aged 3xTg‐AD mice

**DOI:** 10.1002/alz70855_106518

**Published:** 2025-12-24

**Authors:** Juana Andreo‐Lopez, Miriam Bettinetti‐Luque, Cynthia Campos‐Moreno, Celia Da Cunha, Laura Trujillo‐Estrada, Juan Antonio Garcia Leon, Mario Morales‐Cabello, Cristina Nuñez‐Diaz, Alessandra Cadete Martini, Stefania Forner, Antonia Gutierrez, Frank M LaFerla, David Baglietto‐Vargas

**Affiliations:** ^1^ University of Malaga/CIBERNED/IBIMA, Málaga, Spain; ^2^ University of California, Irvine, Irvine, CA, USA

## Abstract

**Background:**

Alzheimer's disease (AD) is a progressive neurodegenerative proteinopathy characterized by the accumulation of amyloid‐beta (Aβ) plaques and tau tangles, leading to synaptic dysfunction and cognitive decline. Increasing evidence suggests that Aβ behaves in a prion‐like manner, propagating misfolded aggregates across the brain. This mechanism involves the seeding and templated misfolding of Aβ, contributing to disease progression. The nature of amyloid seeds, their origin, time of incubation and the host environment, influence aggregation dynamics, yet these factors’ contribution remains poorly known. Recent data have shown that the Aβ source is a key aspect in this process, since its pathogenicity is different according to its origin. Furthermore, recent evidence suggests that microglia plays a key role in the amyloidogenic event, therefore more studies to understand this need to be performed. In this study, we investigated the differential effects of amyloid seeds derived from human AD brain and transgenic mouse models on Aβ and tau propagation, and the inflammatory response.

**Method:**

Amyloid seeds from AD patient (stage C for amyloid; from the Alzheimer's Disease Research Center at UCI) and 25 mo‐3xTg‐AD mice were injected into the hippocampus of 7‐8‐month‐old 3xTg‐AD mice. They were analyzed 10 months post‐surgery for amyloid and microglia markers.

**Result:**

Our findings reveal that human‐derived seeds exhibit a different aggregation pattern compared to mouse‐derived seeds, specifically, triggering the formation of more amyloid aggregates and less neurofibrillary tangles. Moreover, human and mice seeds differentially affect the presence of plaque‐associated microglia in 3xTg‐AD mice, driving the formation of more neuritic pathology in the 3xTg‐AD mice treated with mice seeds.

**Conclusion:**

Our findings suggest that human‐derived amyloid seeds exhibit greater aggregation potential and trigger a stronger inflammatory response than mouse‐derived seeds. Understanding these mechanisms may aid in developing targeted therapies to mitigate amyloid propagation in Alzheimer's disease.

**Funding**: This study was supported by Minister of Science and Innovation grant PID2019‐108911RA‐100 (D.B.V.), Alzheimer's Association grant AARG‐22‐928219 (D.B.V), Beatriz Galindo programBAGAL18/00052 (D.B.V.) and Institute of Health Carlos III (ISCiii) grant PI18/01557 (A.G.) co‐financed by FEDER funds from European Union.

